# Inactivation of TIF1γ Cooperates with Kras^G12D^ to Induce Cystic Tumors of the Pancreas

**DOI:** 10.1371/journal.pgen.1000575

**Published:** 2009-07-24

**Authors:** David F. Vincent, Kai-Ping Yan, Isabelle Treilleux, Fabien Gay, Vanessa Arfi, Bastien Kaniewsky, Julien C. Marie, Florian Lepinasse, Sylvie Martel, Sophie Goddard-Leon, Juan L. Iovanna, Pierre Dubus, Stéphane Garcia, Alain Puisieux, Ruth Rimokh, Nabeel Bardeesy, Jean-Yves Scoazec, Régine Losson, Laurent Bartholin

**Affiliations:** 1 INSERM, U590, IFR62, Lyon, France; 2 Univ Lyon, Lyon, France; 3 INSERM “Avenir” group, Lyon, France; 4 IGBMC, Strasbourg, Illkirch-Cedex, France; 5 Centre Léon Bérard, Lyon, France; 6 INSERM, U865, Faculté Laennec, Lyon, France; 7 INSERM, U758, Lyon, France; 8 Hospices Civils de Lyon, Hôpital Edouard Herriot, Lyon, France; 9 INSERM, U624, Marseille, France; 10 EA 2406, Univ Bordeaux, France; 11 Massachusetts General Hospital Cancer Center, Harvard Medical School, Boston, Massachusetts, United States of America; Fred Hutchinson Cancer Research Center, United States of America

## Abstract

Inactivation of the Transforming Growth Factor Beta (TGFβ) tumor suppressor pathway contributes to the progression of Pancreatic Ductal AdenoCarcinoma (PDAC) since it is inactivated in virtually all cases of this malignancy. Genetic lesions inactivating this pathway contribute to pancreatic tumor progression in mouse models. Transcriptional Intermediary Factor 1 gamma (TIF1γ) has recently been proposed to be involved in TGFβ signaling, functioning as either a positive or negative regulator of the pathway. Here, we addressed the role of TIF1γ in pancreatic carcinogenesis. Using conditional *Tif1γ* knockout mice (*Tif1γ^lox/lox^*), we selectively abrogated *Tif1γ* expression in the pancreas of *Pdx1-Cre;Tif1γ^lox/lox^* mice. We also generated *Pdx1-Cre;LSL-Kras^G12D^;Tif1γ^lox/lox^* mice to address the effect of *Tif1γ* loss-of-function in precancerous lesions induced by oncogenic *Kras^G12D^*. Finally, we analyzed *TIF1γ* expression in human pancreatic tumors. In our mouse model, we showed that *Tif1γ* was dispensable for normal pancreatic development but cooperated with *Kras* activation to induce pancreatic tumors reminiscent of human Intraductal Papillary Mucinous Neoplasms (IPMNs). Interestingly, these cystic lesions resemble those observed in *Pdx1-Cre;LSL-Kras^G12D^;Smad4^lox/lox^* mice described by others. However, distinctive characteristics, such as the systematic presence of endocrine pseudo-islets within the papillary projections, suggest that SMAD4 and TIF1γ don't have strictly redundant functions. Finally, we report that TIF1γ expression is markedly down-regulated in human pancreatic tumors by quantitative RT–PCR and immunohistochemistry supporting the relevance of these findings to human malignancy. This study suggests that TIF1γ is critical for tumor suppression in the pancreas, brings new insight into the genetics of pancreatic cancer, and constitutes a promising model to decipher the respective roles of SMAD4 and TIF1γ in the multifaceted functions of TGFβ in carcinogenesis and development.

## Author Summary

Inactivation of the TGFβ tumor suppressor pathway contributes to the progression of Pancreatic Ductal AdenoCarcinoma (PDAC), a devastating malignancy. Transcriptional Intermediary Factor 1γ (TIF1γ) has recently been proposed to be involved in TGFβ signaling, a pathway inactivated in virtually all cases of this malignancy. To address the role of TIF1γ in pancreatic carcinogenesis, we used conditional *Tif1γ* knockout mice. In a genetic background expressing a constitutively active mutation of *KRAS* oncogene (*Kras^G12D^*) recurrently found in patients with PDAC, *Tif1γ* inactivation induces pancreatic precancerous lesions resembling those observed in the absence of *Smad4*, a key player involved TGFβ signal transduction. This observation strengthens the notion that TIF1γ plays an active role in TGFβ signaling. Interestingly, we also found that TIF1γ expression was markedly down-regulated in human pancreatic tumors supporting the relevance of our findings to human malignancy. Characterization of new players involved in the outbreak of early pancreatic lesions that will eventually evolve into invasive pancreatic cancer is crucial to detect the disease earlier and eventually develop new therapeutic drugs.

## Introduction

Pancreatic Ductal AdenoCarcinoma (PDAC), characterized by a ductal cell-type differentiation pattern, is the most common type of pancreatic cancer, accounting for more than 85% of pancreatic neoplasms. PDAC is the fourth leading cause of cancer-related mortality and carries an overall 5-year-survival rate of less than 5% [1]. The poor outcome of these patients is due to late diagnosis and resistance to current therapies. PDAC appears to arise from precursor lesions known as Pancreatic Intraepithelial Neoplasia (PanINs) or from two types of cystic tumors: Mucinous Cystic Neoplasms (MCNs) and Intraductal Papillary Mucinous Neoplasms (IPMNs) [2]. Mucinous cystic neoplasms are cysts lined by mucin-producing epithelial cells usually associated with an ovarian-type of stroma. These cysts do not communicate with the larger pancreatic ducts. IPMNs form intraductal papillary projections replacing the normal duct epithelium, secrete mucin, and communicate with ducts. IPMNs are currently classified according to their pattern of apparent histological differentiation into three main subtypes: intestinal (with the neoplastic epithelium resembling the intestinal epithelium), the most frequent, pancreatobiliary and gastric [3].

Recurrent genetic alterations have been identified in human PDAC [4],[5]. Sporadic cases, which represent the vast majority of PDAC, are associated with activation of the *KRAS* oncogene (>90% of cases) and inactivation of the *INK4A/ARF* (>80% of cases), *TP53* (>50% of cases) and *SMAD4/DPC4* (>50% of cases) tumor suppressors. Inherited pancreatic cancers represent approximately 5–10% of all pancreatic cancers. In a high proportion of familial pancreatic cancers, the genetic alterations causing the disease are still unknown. However, several germinal mutations associated with complex familial syndromes have been shown to significantly increase the risk of developing pancreatic cancer (*BRCA2*, *INK4A*, *STK11/LKB1*, *PRSS1*, *hMLH1* and *hMSH2*) [1],[6].

In the last five years, a series of genetically engineered mouse models of PDAC have been developed based on these signature gene mutations [7]. For instance, expression of a constitutively active Kras mutant protein (Kras^G12D^ or Kras^G12V^) induces PanINs that eventually progress towards PDAC [8],[9],[10]. Kras activated mutants act in concert with inactivation of the p53 [11], Ink4A/Arf [12],[13], and TβRII [14] tumor suppressors to accelerate development of PDAC. These models and others [15]–[19] support the concept that progression towards invasive PDAC involves emergence from different precancerous lesions (PanINs, MCNs and IPMNs) depending on the associated genetic alterations.

The TGFβ pathway appears to be of particular importance to PDAC tumor suppression, since it is inactivated in virtually all cases of this malignancy [20], and since genetic lesions inactivating the pathway—inactivation of *Smad4* or *TβRII* and over-expression of inhibitory *Smad7*— contribute to pancreatic tumor progression in mouse models [14], [21]–[24]. Transforming growth factor beta (TGFβ) is a secreted polypeptide belonging to a wide family of cytokines and growth factors including TGFβs, Bone Morphogenetic Proteins (BMPs) and activins [25],[26]. Upon binding to its receptors, TGFβ triggers phosphorylation of the SMAD2 and SMAD3 transcription factors. Phosphorylated SMAD2 and SMAD3 then interact with SMAD4. The SMAD2/3/4 complex accumulates within the nucleus, binds to DNA and activates the transcription of target genes leading to proliferative arrest or apoptosis of epithelial cells.

Transcriptional Intermediary Factor 1 gamma (also named TIF1γ/TRIM33/RFG7/PTC7/Ectodermin) [27],[28] appears to contribute to TGFβ signaling, although its precise functional role is not clear. Some data point toward TIF1γ as a negative regulator of the pathway through its capacity to mono-ubiquinate SMAD4 and limit SMAD4 nuclear accumulation [29],[30],[31]. In contrast, other studies have suggested that TIF1γ plays an important positive role in transducing TGFβ signaling through its interaction with SMAD2 and SMAD3 [32].

Here we wished to determine whether TIF1γ contributes to tumorigenesis consistent with a function within the TGFβ signaling pathway. We have focused on pancreatic exocrine tumors based on the prominent role played by TGFβ signaling in these malignancies. Using a conditional mouse strain, we show for the first time that *Tif1γ* is an important gene whose loss of function cooperates with *Kras^G12D^* activation to induce cystic pancreatic tumors resembling human IPMNs. We also report that *TIF1γ* expression is down-regulated in human PDAC and some types of precursor lesions, supporting the relevance of our mouse model to human malignancy.

## Results/Discussion

To selectively abrogate *Tif1γ* expression in the pancreas, we crossed conditional *Tif1*γ knockout mice [33] with *Pdx1-Cre* mice [34]. *Pdx1* is a gene expressed in the common progenitor to all pancreatic lineages during early embryogenesis, hence *Pdx1-Cre* transgenic mice exhibit recombination of floxed alleles in pancreatic cells from all lineages (endocrine, acinar, centroacinar and ductal cells) [35]. *Pdx1-Cre;Tif1γ^lox/lox^* animals were born at expected ratios and showed normal lifespan without obvious developmental or physiological alterations. Live imaging techniques (Positron Emission Tomography, PET and Magnetic Resonance Imaging, MRI), histological techniques (immunodetection of insulin, glucagon, PPY, chymotrypsine, F4/80, CD3, MPO), metabolic tests (glucose tolerance) did not reveal any significant differences between wild-type and *Pdx1-Cre;Tif1γ^lox/lox^* littermates (n>20, between 3 weeks and 2 years of age) (data not shown). As expected, immunohistochemistry experiments showed that Tif1γ was expressed in the nuclei of pancreatic cells in wild-type mice and that this staining was lost in the *Pdx1-Cre;Tif1γ^lox/lox^* pancreas (Figure S1). In all, these observations show that *Tif1γ* is dispensable for normal pancreatic development and function in the mouse.

**Figure 1:**
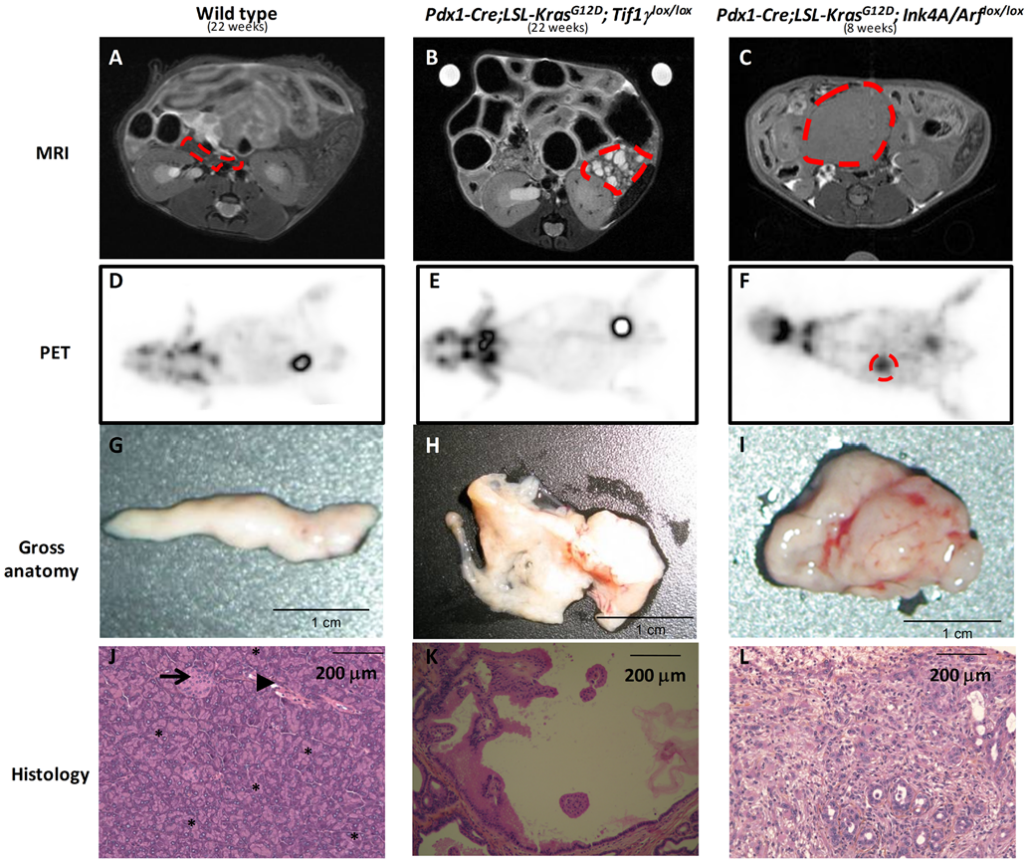
Homozygous deletion of *Tif1γ* cooperates with activated *Kras^G12D^* mutation to induce cystic tumors of the pancreas. Magnetic Resonance Imaging (MRI) (A–C). Positron Emission Tomography (PET) (D–F). Gross anatomy of the pancreas after dissection (G–I). Hematoxylin Phloxine Saffron (HPS) staining of pancreas sections (J–L). Acinar tissue (stars), scattered endocrine islets (arrow) and exocrine ducts (arrowhead). When notified, pancreas is circled in red dashed lines. *Pdx1-Cre;LSL-Kras^G12D^;Tif1γ^lox/lox^* used in this experiment was 154 days old and representative of the 4 mice we analyzed at the age of 18–27 weeks.

Activating *KRAS* mutations occur early in human PDAC pathogenesis and give rise to slowly progressing PanINs in mouse models [8],[10],[12]. We then asked whether *Tif1γ* inactivation could modify the phenotype or latency of the pancreatic lesions induced by *Kras^G12D^*. To that end, we generated *Pdx1-Cre;LSL-Kras^G12D^;Tif1γ^lox/lox^* mice (n = 12, Table S1). All animals looked healthy at the time they were euthanized (the oldest animal was sacrificed at the age of 189 days). Since pancreatic lesions are often asymptomatic, we decided to explore *in vivo* the pancreas of these mutant mice (n = 4) by PET and MRI imaging techniques. *Pdx1-Cre;LSL-Kras^G12D^;Ink4A/Arf^lox/lox^* mice, which exhibit rapid PDAC progression, were also employed in these studies. Strikingly, MRI imaging performed on *Pdx1-Cre;LSL-Kras^G12D^;Tif1γ^lox/lox^* animals revealed an hypertrophic pancreas with multifocal cystic lesions exhibiting T2 hypersignals visible as early as 7 weeks after birth (T2 weighted scans allow detection of cysts as they are sensitive to water content) (Figure 1B). Such lesions were absent in the pancreas of wild-type and *Pdx1-Cre;LSL-Kras^G12D^* animals (Figure 1A) and were clearly different from those observed in *Pdx1-Cre;LSL-Kras^G12D^;Ink4A/Arf^lox/lox^* mice, which harbor solid tumors exhibiting a T1 isosignals (T1 weighed scans allow detection of solid tumors) (Figure 1C). PET imaging did not show significant increased metabolic activity in the abdomen of wild-type (Figure 1D) and *Pdx1-Cre;LSL-Kras^G12D^;Tif1γ^lox/lox^* mice (Figure 1E) whereas *Pdx1-Cre;LSL-Kras^G12D^;Ink4A/Arf^lox/lox^* mice had abdominal lesions with readily detectable metabolic activity (Figure 1F). Macroscopic analysis of the *Pdx1-Cre;LSL-Kras^G12D^;Tif1γ^lox/lox^* pancreas confirmed the presence of numerous cysts affecting the entire organ without macroscopic evidence of invasive carcinoma (Figure 1H and Figure S2) whereas the pancreas of *Pdx1-Cre;LSL-Kras^G12D^;Ink4A/Arf^lox/lox^* mice was invaded by a firm and homogeneous mass (Figure 1I) and the pancreas from wild-type or *Pdx1-Cre;LSL-Kras^G12D^* animals had a normal macroscopic appearance (Figure 1G). The size of these cysts observed in the pancreas from *Pdx1-Cre;LSL-Kras^G12D^;Tif1γ^lox/lox^* was variable and most of them contained papillary projections (Figure 1K). These lesions clearly contrast with the invasive tumors of ductal morphology identified as PDAC in the pancreas of *Pdx1-Cre;LSL-Kras^G12D^;Ink4A/Arf^lox/lox^* (Figure 1L). The histological analysis of the 12 *Pdx1-Cre;LSL-Kras^G12D^;Tif1γ^lox/lox^* animals (Table S1) revealed the presence of cystic lesions in 100% of these mice, such cysts being never observed in *Pdx1-Cre;LSL-Kras^G12D^* or wild type control animals. Quantitative analysis revealed that the area occupied by the abnormal pancreas exceeded 50% by the age 6 weeks in 6/7 *Pdx1-Cre;LSL-Kras^G12D^;Tif1γ^lox/lox^* mice whereas it represented less than 20% in 6/6 *Pdx1-Cre;LSL-Kras^G12D^* mice (Table S1). Collectively these data demonstrate that inactivation of *Tif1γ* actively cooperates with activated *Kras^G12D^* to induce cystic tumors of the pancreas.

**Figure 2:**
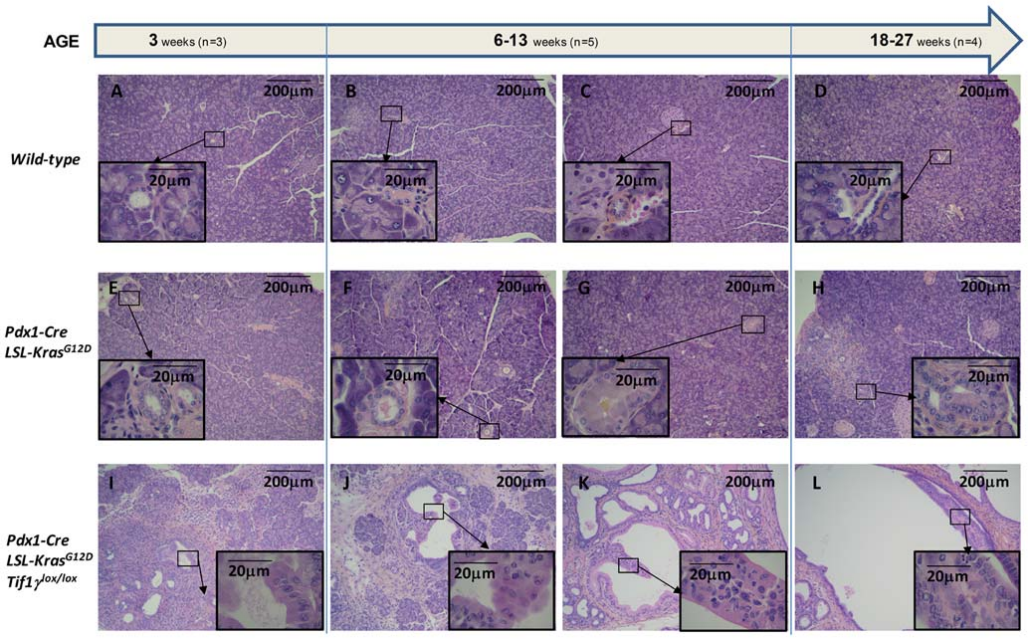
Inactivation of *Tif1γ* accelerates activated *Kras^G12D^*–mediated pancreatic neoplasia. Sections of wild-type (A–D), *Pdx1-Cre;LSL-Kras^G12D^* (E–H) and *Pdx1-Cre;LSL-Kras^G12D^;Tif1γ^lox/lox^* (I–L) pancreas from mice at different ages were stained with HPS. Number of mice: 3 weeks, n = 3; 6–13 weeks, n = 5; 18–27 weeks, n = 4. Inset pictures: Higher magnification.

To carefully compare the pancreatic lesions observed in *Pdx1-Cre;LSL-Kras^G12D^;Tif1γ^lox/lox^* with the *Pdx1-Cre;LSL-Kras^G12D^* controls, we performed a sequential histological analysis of pancreas from animals (n = 12) euthanized at different ages (Table S1). Contrary to wild-type mice (Figure 2A–2D), *Pdx1-Cre;LSL-Kras^G12D^* mice gradually developed focal PanINs by the age of about 10 weeks (Figure 2E–2H). Strikingly, in *Pdx1-Cre;LSL-Kras^G12D^;Tif1γ^lox/lox^* pancreas, PanINs, signs of acute inflammation as well as enlarged and dilated ductal structure resembling budding cysts were observed as early as 3 weeks of age (Figure 2I and Figure S3). At later time points, inflammatory tissue and PanINs were mainly replaced with cystic lesions becoming more numerous and of larger size (Figure 2J–2L). Microscopic examination revealed that the lining of the cystic structures characteristically found in the pancreas from *Pdx1-Cre;LSL-Kras^G12D^;Tif1γ^lox/lox^* mice was formed by epithelial cells with a cuboidal or cylindrical morphology. These cells formed numerous thick papillary projections in the cyst lumen. The axis of these projections usually contained masses of small monomorphic cells with an endocrine morphology (Insets in Figure 2I–2L).

**Figure 3:**
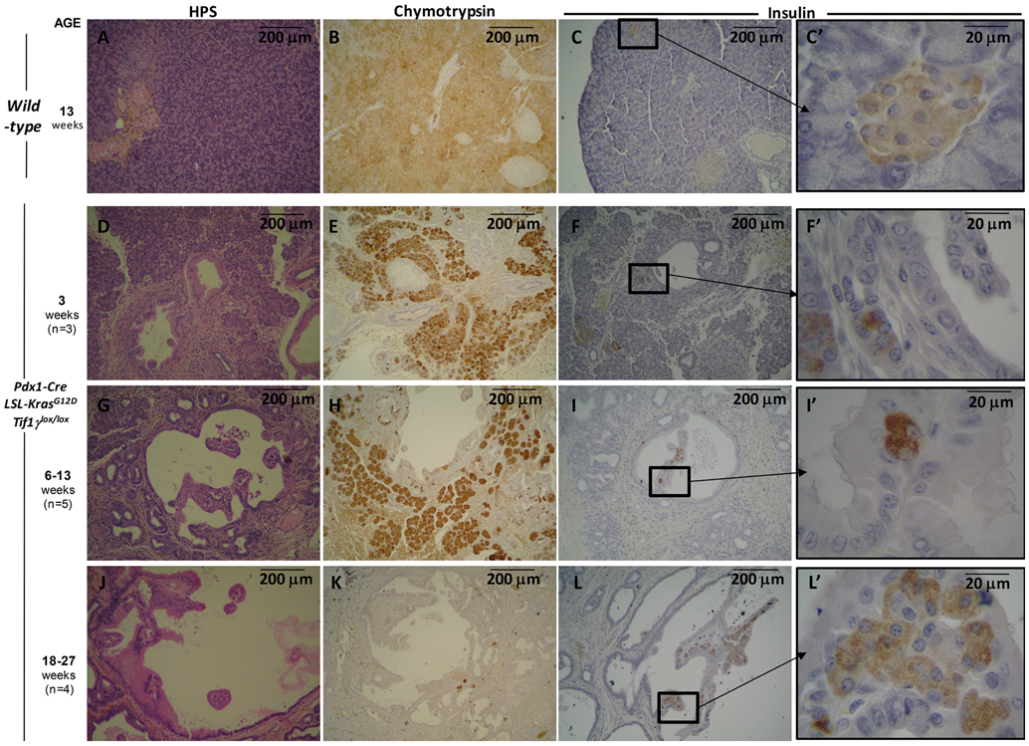
Endocrine and exocrine compartments destruction in the pancreas of *Pdx1-Cre;LSL-Kras^G12D^;Tif1γ^lox/lox^* mice. Wild-type (A–C′) and *Pdx1-Cre; LSL-Kras^G12D^; Tif1γ^lox/lox^* (D–L′) pancreas sections were stained with HPS (A,D,G,J), immuno-revealed either with an anti-chymotrypsin antibody (B,E,H,K) or an anti-insulin antibody (C,F,I,L and C′,F′,I′,L′ that represent the boxed region in C,F,I,L at a higher magnification). For *Pdx1-Cre;LSL-Kras^G12D^;Tif1γ^lox/lox^* mice, analysis were performed at different ages. Number of mice: 3 weeks, n = 3; 6–13 weeks, n = 5; 18–27 weeks, n = 4. Boxes: Higher magnification. The pictures show one representative mouse for each group.

We performed immunohistochemical studies to characterized the evolving pancreatic lesions in these mice. We observed staining for chymotrypsin and insulin, which decreased with age, indicating a replacement of exocrine and endocrine components, together with abnormal ductal structures in *Pdx1-Cre;LSL-Kras^G12D^;Tif1γ^lox/lox^* mice (Figure 3). There was a notable disappearance of well-organized endocrine islets with age coinciding with the accumulation of endocrine cells within the papillary projections bulging within the lumen of the cysts.

**Figure 4:**
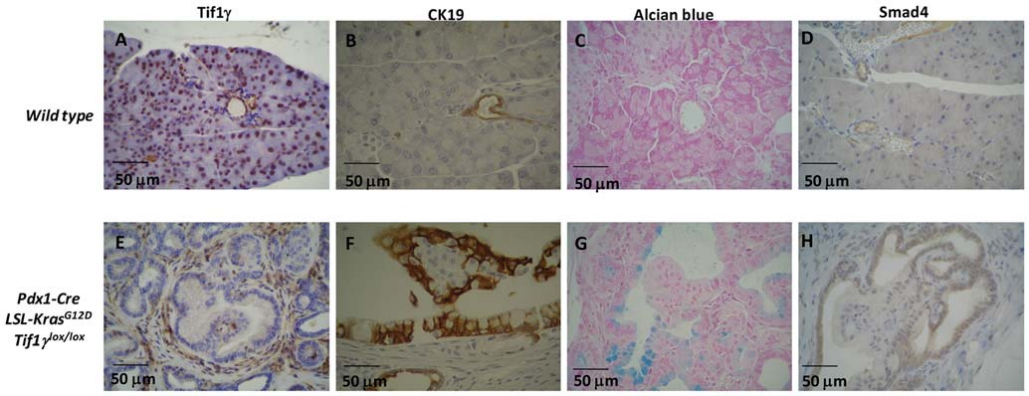
Immunophenotyping of pancreas cysts observed in *Pdx1-Cre;LSL-Kras^G12D^;Tif1γ^lox/lox^* mice. Wild-type (A–D) and *Pdx1-Cre;Tif1γ^lox/lox^;LSL-Kras^G12D^* (E–H) pancreas sections were stained to detect glandular cells (CK19), mucus-secreting cells (Alcian Blue), Tif1γ and Smad4. The pictures show one representative mouse at the age of 22 weeks.

To more precisely identify the nature of these lesions, several lineage markers were explored by immunohistochemistry. We first verified that Tif1γ expression was lost in pancreatic ducts from *Pdx1-Cre;LSL-Kras^G12D^;Tif1γ^lox/lox^* mice (Figure 4E) compared to normal observed ducts in wild-type mice (Figure 4A). In the normal pancreas, cytokeratin 19 (CK19) is specifically expressed by ductal cells lining the secretory ducts (Figure 4B). We verified that most of the epithelial cells lining the cystic lesions observed in *Pdx1-Cre;LSL-Kras^G12D^;Tif1γ^lox/lox^* mice were positive for CK19 (Figure 4F); this is consistent with a ductal phenotype for these cells. However, in contrast to the cells lining the normal secretory ducts (Figure 4C), many cells lining the cysts were mucus-secreting and stained for Alcian blue (Figure 4G). The cells with an endocrine appearance present within the intra-cystic papillary projections were CK19 and Alcian blue negative. There was no evidence of ovarian-type stroma or of invasive or microinvasive carcinoma, even on serial sections, suggesting that these cystic tumors resemble human IPMNs. In all, the cystic lesions observed in *Pdx1-Cre;LSL-Kras^G12D^;Tif1γ^lox/lox^* mice show distinctive characteristics, including the presence of intra-epithelial endocrine pseudo-islets, suggestive of a mixed, endocrine-exocrine, lesion [7].

TIF1γ has recently been proposed to be involved in TGFβ signaling [29],[32]. The resemblance between the cystic lesions (either IPMNs [21],[23] or MCNs [22]) observed in *Pdx1-Cre;LSL-Kras^G12D^;Smad4^lox/lox^* mice, and the cystic lesions we observed in the *Pdx1-Cre;LSL-Kras^G12D^;Tif1γ^lox/lox^* mice, reinforces an active role of TIF1γ in TGFβ signaling. However, we cannot rule out the possibility that TIF1γ could also be involved in other signaling pathways. Interestingly, we observed that Smad4 expression was almost undetectable in epithelial cells lining the papillary projections observed in *Pdx1-Cre;LSL-Kras^G12D^;Tif1γ^lox/lox^* mice whereas it was detectable in epithelial cells lining the cysts (Figure 4H) or in normal ducts (Figure 4D). IPMNs observed in *Pdx1-Cre;LSL-Kras^G12D^;Tif1γ^lox/lox^* mice always contain a significant endocrine component, a rare event in IPMNs observed in *Pdx1-Cre;LSL-Kras^G12D^;Smad4^lox/lox^* mice [21],[23]. This observation suggests that TIF1γ and SMAD4 could differentially regulate endocrine versus exocrine differentiation in a context of an activated KRAS oncogenic protein.

**Figure 5:**
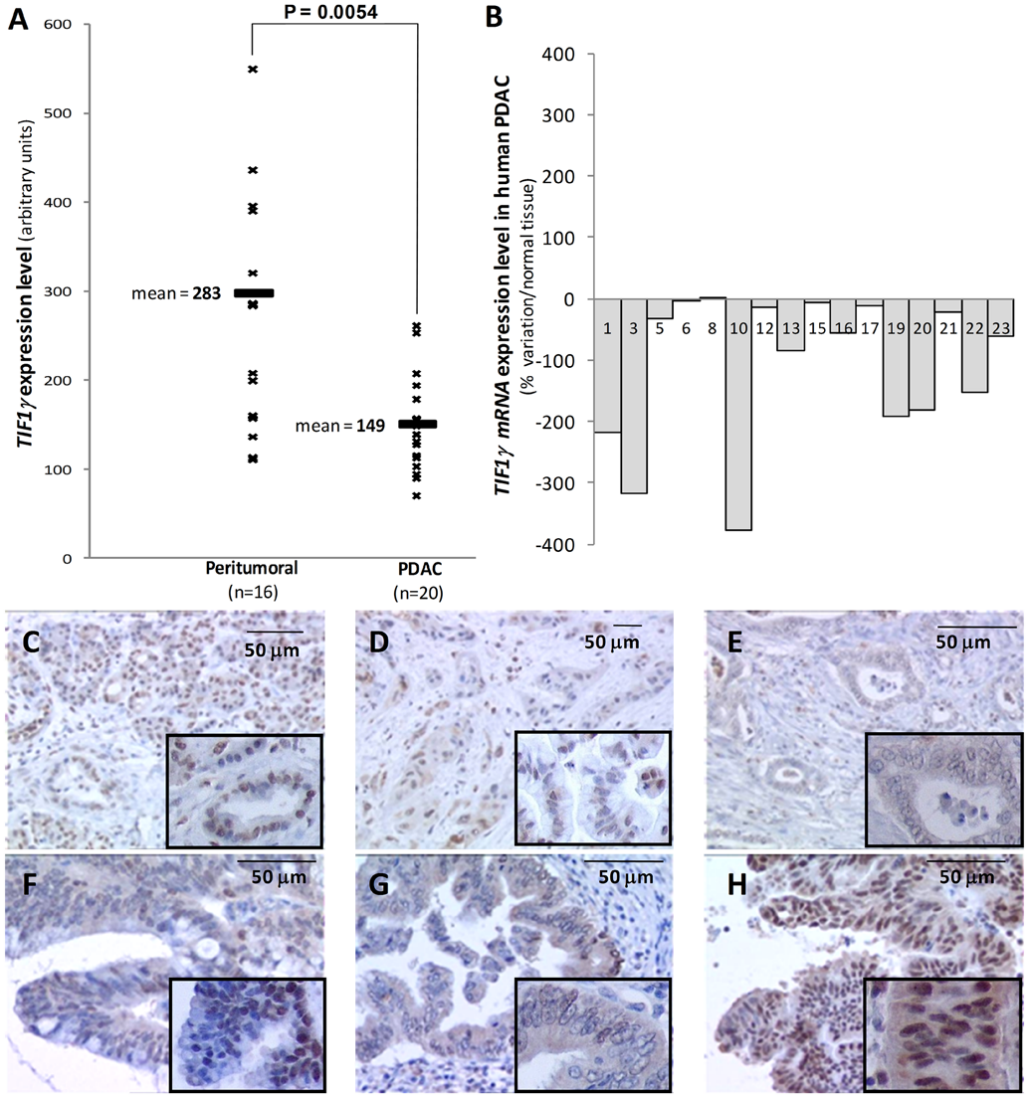
*TIF1γ* expression in human pancreatic neoplasia. The expression of *TIF1γ* from 20 human PDAC and 16 peritumoral tissues was determined by quantitative RT–PCR (A). The average expression (±sd) is plotted for each group (B). The significance (P value) for the difference between peritumoral and tumoral groups, determined by a Student's T test, is shown above the graphs. The expression of TIF1γ protein was evaluated by immunohistochemistry in different pancreatic lesions: PDAC (n patients = 20) (peritumoral (C) and tumoral tissues (D, E). IPMN (n patients = 10) of high grade dysplasia (F), high-grade PanINs (n patients = 8) (G) and MCN (n patients = 15) (H). Inset pictures: higher magnification.

Based on the prominent cooperation noted between *Kras* activation and *Tif1γ* inactivation in promoting cystic pancreatic tumors in our mouse model, we speculated that *TIF1γ* expression may be down-regulated in human pancreatic tumors. To test this hypothesis, we analyzed by quantitative RT-PCR the expression level of *TIF1γ* and *SMAD4* mRNA in 20 PDAC and 16 peritumoral tissues coming from surgical specimens removed for therapeutic purposes (peritumoral tissues were not available for 4 of these patients). The cellularity of the samples used for molecular analysis was verified histologically. Our results show that *TIF1γ* expression is significantly decreased in the tumors as compared to peritumoral tissues (*P* = 0.0054) (Figure 5A). We also compared *TIF1γ* expression levels in each individual tumor along with the peritumoral tissue from the same patient (n = 16). Our results show that *TIF1γ* expression is significantly down-regulated in most patients and is not up-regulated in any patient (Figure 5B). We next examined TIF1γ protein pattern of expression by immunohistochemistry in human pancreatic cancers and their precursors. In peritumoral tissues from PDAC, TIF1γ was detected in the majority of the nuclei of acinar, ductal and endocrine cells (Figure 5C). Centroacinar cells are more difficult to identify in routinely stained sections; however, since no epithelial cell population devoid of TIF1γ expression has been detected in the normal pancreas, it can be assumed that they also express TIF1γ. In PDAC (16 cases), TIF1γ nuclear expression level was significantly decreased as compared to the peritumoral tissue. In 8 cases, TIF1γ expression was heterogeneous, with large numbers of negative cells coexisting with scattered positive cells (Figure 5D). In 2 cases, TIF1γ was even undetectable (Figure 5E). In IPMNs (samples from 10 patients, all with the intestinal subtype according to current classifications [3]), almost all neoplastic cells in areas of low grade dysplasia (present in the 10 cases) displayed a weak nuclear positivity whereas in areas of high grade dysplasia (present in the 10 cases), more than 50% of cells were negative for TIF1γ; this was especially the case along the papillary projections (Figure 5F). In PanINs (samples from 15 patients, with grade 1 in 12, grade 2 in 10 and grade 3 in 8), the expression of TIF1γ was usually retained in grade 1 and 2 lesions (data not shown), but was undetectable in a variable proportion of cells in grade 3 lesions (Figure 5G). In MCNs (8 cases), TIF1γ protein was strongly expressed by all neoplastic cells, even in areas of high grade dysplasia and in foci of microinvasive carcinoma (Figure 5H). We showed in the same set of tumors that *SMAD4* expression was also down-regulated in high-grade PanINs, IPMNs and PDAC, while remained highly expressed in MCNs (Figure S4).

None of the *Pdx1-Cre;LSL-Kras^G12D^;Tif1γ^lox/lox^* mice (n = 4) sacrificed after the age of 13 weeks showed an aggressive cancer developed from IPMNs. This observation is consistent with epidemiological data in humans showing that IPMNs only rarely give rise to aggressive tumors [3]. Interestingly, IPMNs were reported in *Pdx1-Cre;LSL-Kras^G12D^;Smad4^lox/lox^* mice but no PDAC was found by the age of 13 weeks (n = 8) [21]. Another group observed in *Pdx1-Cre;LSL-Kras^G12D^;Smad4^lox/lox^* a significant proportion of PDAC between 23 and 33 weeks [23]. This suggests that the minimal latency period to see the onset of aggressive tumors may have not been reached in our study or the number of animals studied is too low. We have been in the process of “aging” a cohort of *Pdx1-Cre;LSL-Kras^G12D^;Tif1γ^lox/lox^* mice to address this specific point even if we cannot rule out the possibility that these animals die before developing aggressive tumors because of pancreatic failure due to growing cysts. The molecular mechanism supporting the cooperation between activated *Kras^G12D^* mutation and *Tif1γ* inactivation to induce the formation of IPMNs is still unknown. TAK1 (TGFβ Associated Kinase 1), which has recently been proposed to explain R-Ras and TGFβ cooperation in breast tumors, is an interesting candidate [36].

The relationship between TIF1γ and SMAD4 and their respective role in TGFβ signaling have been a subject of extensive investigation and debate in the last few years [37]. Indeed, published data support distinct models whereby TIF1γ could function as either a negative regulator of TGFβ signaling [29],[30] or a complementary agonist of TGFβ signaling [32]. In the “antagonist model”, TIF1γ negative function relies on its ability to mono-ubiquitinate and relocate SMAD4 into the cytoplasm. In the “agonist model”, TIF1γ competes with SMAD4 for binding to SMAD2 and 3 and form TIF1γ-SMAD2/3 complexes regulating SMAD4-independent TGFβ responses. One can envision that these models, both supported by compelling biochemical and *in vivo* evidence, are not mutually exclusive and that one of them may be predominant depending on the cellular context. The experimental evidence we present here suggest that TIF1γ works with SMAD4 as a complementary agonist molecule during pancreatic tumorigenesis. In the presence of activated Kras, Tif1γ loss-of-function induces cystic lesions resembling those observed in the absence of Smad4 suggesting that both molecules act in concert to prevent tumor progression. Even if this hypothesis needs further demonstration, it is strengthened by the observation that TIF1γ expression is decreased in human pancreatic tumors and our observation that loss of Tif1γ does not significantly impair Smad4 expression level or *Smad4* target genes expression (data not shown). The existence of a joint effort between TIF1γ and SMAD4 to maintain TGFβ-mediated tissue homeostasis has been proposed before. Indeed, during erythroid differentiation, TIF1γ mediates the differentiation response while SMAD4 mediates the antiproliferative response [32]. In a recent work, we showed that Tif1γ controlled iNKT (invariant Natural Killer T) cell expansion whereas Smad4 maintained their maturation state [33]. A recent comprehensive genetic analysis of >20,000 transcripts in 24 pancreatic cancers failed to identify point mutations, amplifications, deletion or translocations in the *TIF1γ* gene [20]. The present study strongly spurs us toward looking for *TIF1γ* genetic alterations in a larger set of pancreatic tumors. Besides, chromosomal breakpoints chromosome on 1p13.1 containing *TIF1γ* gene have been reported in acute megakaryocytic leukemias [38], osteochondromas [39], bronchial large cell carcinomas [40] and childhood papillary thyroid carcinomas [41]. Interestingly, we recently demonstrated that abrogation of the closely related *Tif1α* gene in mice caused hepatocellular carcinoma [42]. These observations reinforce the idea according to which TIF1γ loss of function could play an active protective role during tumorigenesis. TIF1γ overexpression has been suggested by others to facilitate tumorigenesis in other organs by inhibiting SMAD4-mediated growth inhibition and motility in response to TGFβ [29],[30]. This observation may reflect an active role of TIF1γ during tumor progression depending on the organ and involving a anti-SMAD4 mechanisms (“antagonist” model).

In conclusion, we demonstrated in a mouse model that inactivation of *Tif1γ* cooperates with activated *Kras^G12D^* to induce cystic pancreatic tumors. Characterization of new players involved in the outbreak of early pancreatic lesions that will eventually evolve into invasive pancreatic cancer is crucial to detect the disease earlier and eventually develop new therapeutic drugs. Further work to decipher the respective roles of SMAD4 and TIF1γ in PDAC as well as the functional cooperation between KRAS and TIF1γ could bring new insight into the etiology of pancreatic cancer, and generate a better understanding of the multifaceted role of TGFβ in carcinogenesis and development.

## Materials and Methods

### Mice

*Tif1γ^lox/lox^* [32] mice harboring floxed exons 2–4 were generated by K.Y. and R.L. will be described elsewhere. Briefly, using a genomic clone that contains a portion of the *Tif1*γ gene, we generated a targeting vector in which a *PGK Neo* selection cassette flanked by two *loxP* sites was introduced into intron 1 and a third *loxP* site inserted into intron 4. This targeting vector was designed with the expectation that upon homologous recombination and subsequent Cre recombinase-mediated excision, exons 2, 3 and 4 along with the *PGK-Neo* cassette would be deleted, thereby causing a frameshift mutation with a premature termination codon in exon 5. The putative product of this deleted gene corresponds to a truncated protein lacking part of the RING finger-B box-coiled coil (RBCC) motif and the entire C-terminal region of the TIF1γ protein, which contains the conserved PHD finger/bromodomain unit.

*Ink4A/Arf^lox/lox^* [12], *LSL-Kras^G12D^* [8], [43]–[45] and *Pdx1-Cre* [34] mouse strains done by others were previously described. Mice were maintained in a specific pathogen-free animal facility at the “Centre Léon Bérard” (Lyon, France) and handled in compliance with the institutional guidelines. All procedures were approved by an ethic committee under regulatory of governmental authority (CREEA).

### Histology

Histology experiments were performed as previously described [46]. Primary antibody used were : TIF1γ (1/800; Euromedex), Chymotrypsin (1/1000; AbD Serotec), Insulin (1/1000; Dako), CK19 (1/10; Trauma III - Developmental Studies Hybridoma Bank - University of Iowa), SMAD4 (1/100; Santa Cruz). Alcian blue staining was performed as previously described [47].

### Human samples

Cryopreserved tumoral and peritumoral tissue samples were obtained from an institutional tissue bank, the Tumorothèque des Hospices Civils de Lyon (Centre de Ressources Biologiques, Hospices Civils de Lyon). In accordance with French ethical rules, samples were from patients having given their informed consent or from deceased patients. Prior to molecular analysis, the quality and cellularity of tissue samples was verified histologically; tumor tissues were selected in order to contain a significant amount of neoplastic cells; peritumoral tissues were constantly altered by reactive fibrotic changes associated with a loss in acinar tissue and a massive ductular proliferation.

### RNA analysis

Liquid nitrogen frozen human tumors were blended using a Pro200 homogenizer (Pro Scientific Inc.) in a 5 M guanidine solution. Total RNA was further purified by RNeasy mini kit (Qiagen). The cDNA was used as template with RT Kit SuperScript II (Invitrogen). quantitative RT-PCR was performed as previously described [48].

### Live imaging

For MRI and PET experiments, mice were anesthetized using 3% isoflurane inhalation (TEM Sega, Lormont, France) and maintained in 1.5% isoflurane atmosphere during experiments.

For PET experiments, the mice were catherized in the caudal vein (24 gauge), injected with 250 µCi of 300 µL of radioactive 18-Fluorodéoxyglucose (FDG). After 90 minutes to allow FDG fixation, images were acquired during 15 minutes (constant 2% isoflurane atmosphere) using the “TEP clearPET” (Raytest, Inc.). MRI acquisitions were made with a BioSpec-7T system (Bruker, Ettlingen, Germany) using a 32 mm inner-diameter emission/reception volume coil (Rapid Biomedical, Würzburg, Germany). T1/T2-weighted contrast sequences synchronized to respiration were acquired for each mouse. A RARE (Rapid Acquisition with Relaxation Enhancement) sequence (TR/TE 3500/38.1 ms) with fat saturation was used. Geometric parameters were: a series of 18, 750 µm thick sections, 33 mm field of view, and 256×256 pixel matrix. Voxel size was therefore 129×129×750 µm^3^.

## Supporting Information

Figure S1 Loss of nuclear Tif1γ protein expression in the pancreas of *Pdx1-Cre; Tif1γ^lox/lox^* mice. Immunohistochemistry showed that Tif1γ was expressed in the nuclei of pancreatic cells in wild-type mice and that this staining was lost in a *Pdx1-Cre;TIF1γ^lox/lox^* pancreas.
(2.25 MB TIF)

Figure S2 Image of a polycystic pancreas from a 154-day-old *Pdx1-Cre; LSL-Kras^G12D^; Tif1γ^lox/lox^* mouse. Pancreas is circled in black. Note the presence of numerous translucent cysts (arrowhead).
(2.96 MB TIF)

Figure S3 Presence of inflammatory cells in the pancreas associated with the cystic structures. Immunohistochemistry revealed the presence of different populations of leucocytes infiltrating the pancreas of a 20-day-old *Pdx1-Cre; LSL-Kras^G12D^; Tif1γ^lox/lox^* mouse (D–F) compared to a wild-type littermate (A–C). MPO (A,D), F4/80 (B,E), and CD3 (C,F) were respectively used as specific markers for neutrophiles, macrophages and lymphocytes.
(2.71 MB TIF)

Figure S4 *SMAD4* expression in human pancreatic neoplasia. Quantitative RT–PCR to detect *SMAD4* expression from the 16 PDAC for which peritumoral tissue was available (A). For each patient represented by an individual bar, *SMAD4* expression is represented as a percentage of variation relative to mRNA expression in the peritumoral tissue from the same patient (B). SMAD4 protein expression pattern was also assessed by immunohistochemistry. In the normal pancreas (C), SMAD4 is strongly detected in endocrine islets, a faint labeling is visible in acinar cells and ductal cells. In adenocarcinomas (n patients = 20), no labeling for SMAD4 was detected whereas adjacent residual endocrine cells are positive (D). In another example of adenocarcinoma, SMAD4 is faintly but readily detectable in neoplastic cells; the labeling is both cytoplasmic and nuclear (E). In IPMN grade 3, SMAD4 expression is heterogeneous; most cells are negative, while a few scattered cells retain a faint expression, usually nuclear (F). In PanIN grade 3, SMAD4 expression is either undetectable, as exemplified in the largest figure (note the persistent expression in adjacent residual endocrine cells (arrow)), or heterogeneous, as shown in the inset (G). In MCN, SMAD4 expression is usually strong, in low grade (large figure) as well as in high grade (inset) lesions (H).
(3.34 MB TIF)

Table S1 Measurement of the area occupied by the normal pancreatic tissue.
(0.06 MB DOC)
