# Correction: Inactivation of TIF1γ Cooperates with Kras^G12D^ to Induce Cystic Tumors of the Pancreas

**DOI:** 10.1371/annotation/7941c465-532f-4b42-b541-72d0810943b9

**Published:** 2009-08-06

**Authors:** David F. Vincent, Kai-Ping Yan, Isabelle Treilleux, Fabien Gay, Vanessa Arfi, Bastien Kaniewski, Julien C. Marie, Florian Lepinasse, Sylvie Martel, Sophie Goddard-Leon, Juan L. Iovanna, Pierre Dubus, Stéphane Garcia, Alain Puisieux, Ruth Rimokh, Nabeel Bardeesy, Jean-Yves Scoazec, Régine Losson, Laurent Bartholin

The sixth author's name was spelled incorrectly. The correct name is: Bastien Kaniewski.

