# Supplementary figures and images for: Inactivation of TIF1γ Cooperates with KrasG12D to Induce Cystic Tumors of the Pancreas
Source: PLoS Genet. 2009 Jul 24;5(7):e1000575. doi: 10.1371/journal.pgen.1000575 (PMC2706992; doi:10.1371/journal.pgen.1000575)

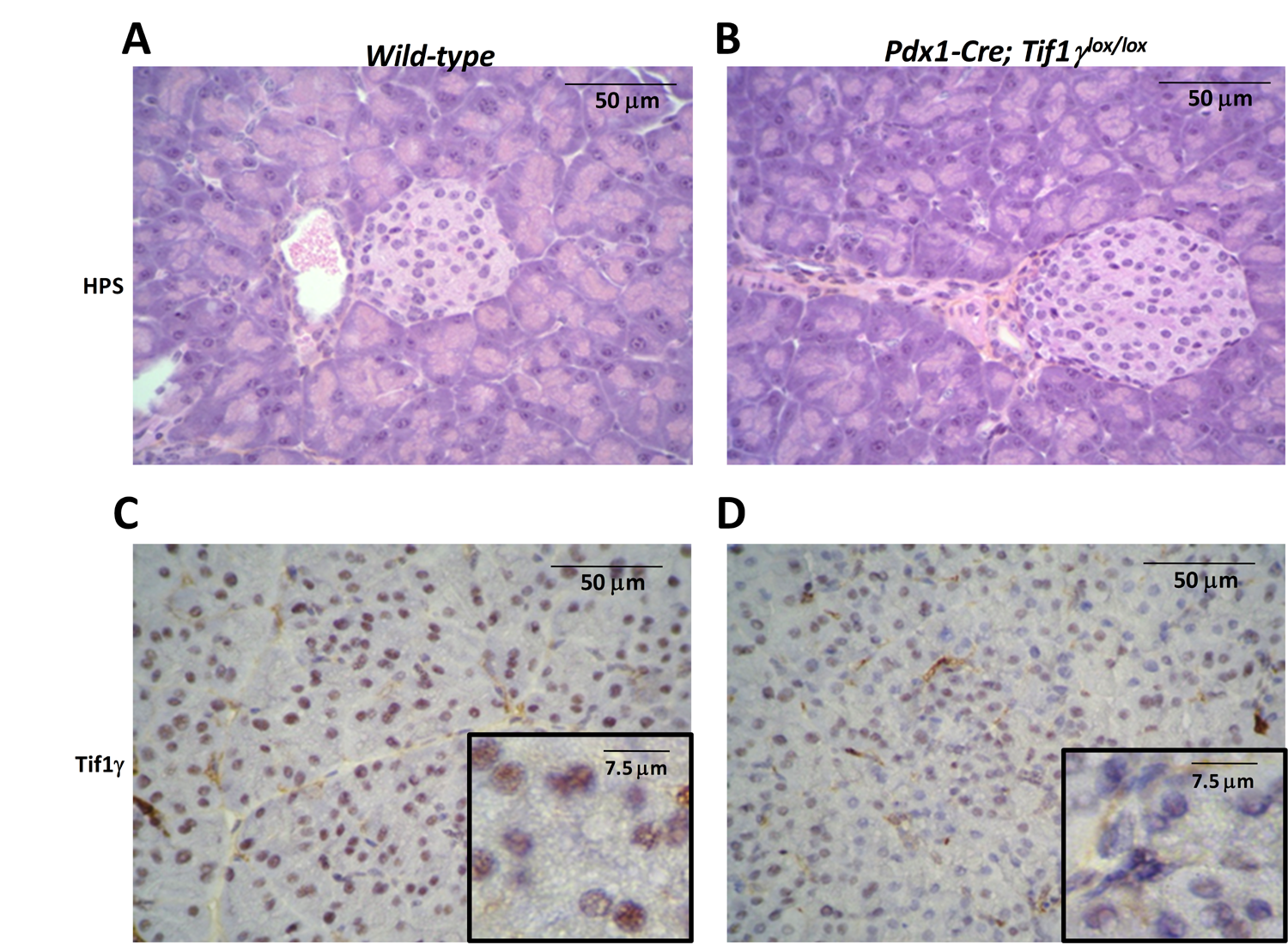

Supplement: Figure S1 — Loss of nuclear Tif1γ protein expression in the pancreas of Pdx1-Cre; Tif1γlox/lox mice. Immunohistochemistry showed that Tif1γ was expressed in the nuclei of pancreatic cells in wild-type mice and that this staining was lost in a Pdx1-Cre;TIF1γlox/lox pancreas. (2.25 MB TIF) [file pgen.1000575.s001.tif]

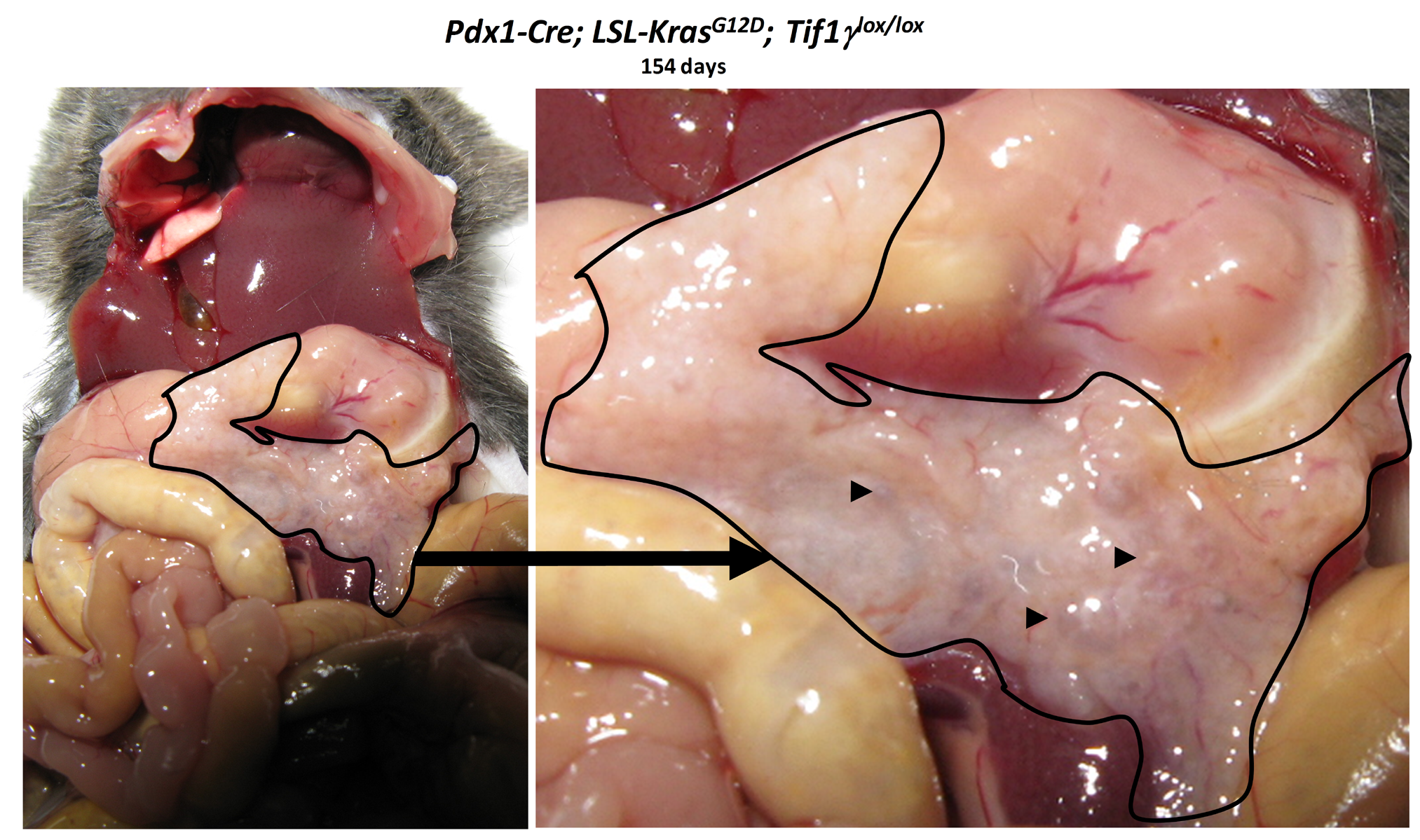

Supplement: Figure S2 — Image of a polycystic pancreas from a 154-day-old Pdx1-Cre; LSL-KrasG12D; Tif1γlox/lox mouse. Pancreas is circled in black. Note the presence of numerous translucent cysts (arrowhead). (2.96 MB TIF) [file pgen.1000575.s002.tif]

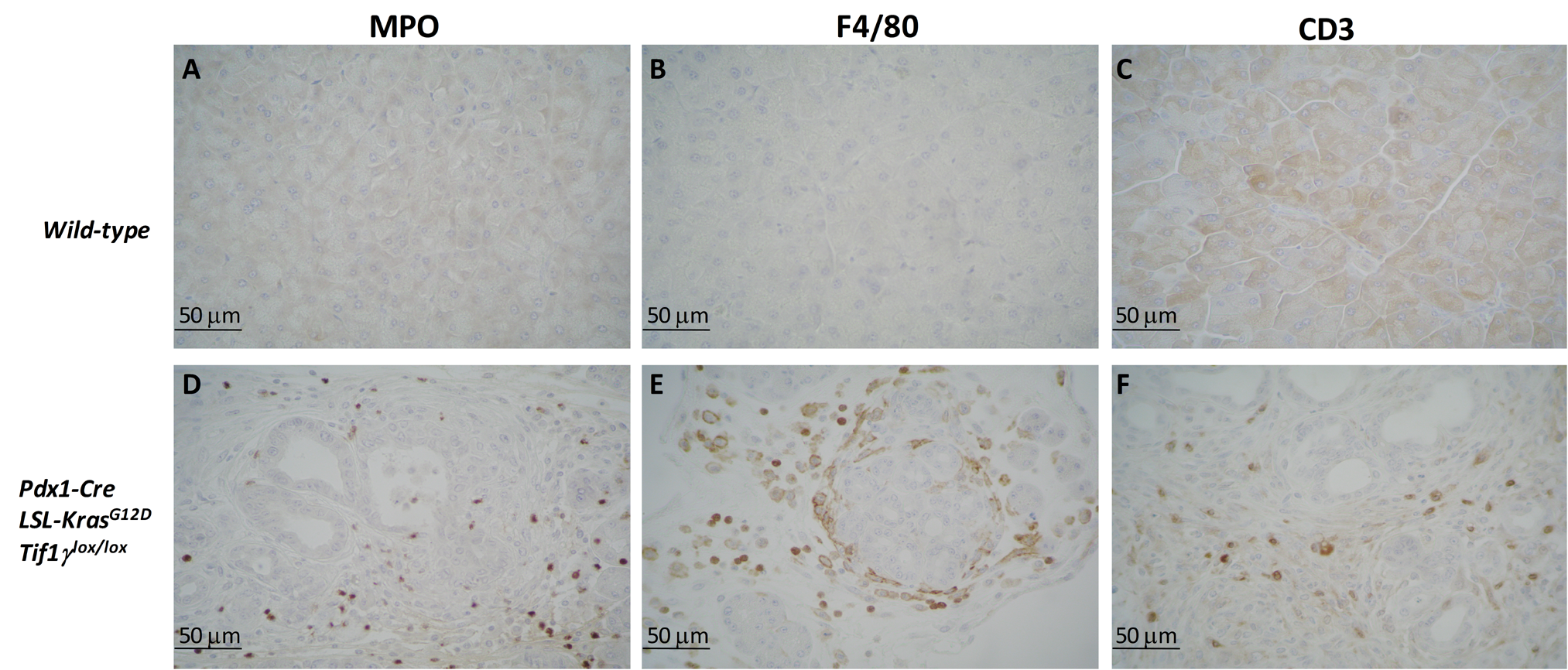

Supplement: Figure S3 — Presence of inflammatory cells in the pancreas associated with the cystic structures. Immunohistochemistry revealed the presence of different populations of leucocytes infiltrating the pancreas of a 20-day-old Pdx1-Cre; LSL-KrasG12D; Tif1γlox/lox mouse (D–F) compared to a wild-type littermate (A–C). MPO (A,D), F4/80 (B,E), and CD3 (C,F) were respectively used as specific markers for neutrophiles, macrophages and lymphocytes. (2.71 MB TIF) [file pgen.1000575.s003.tif]

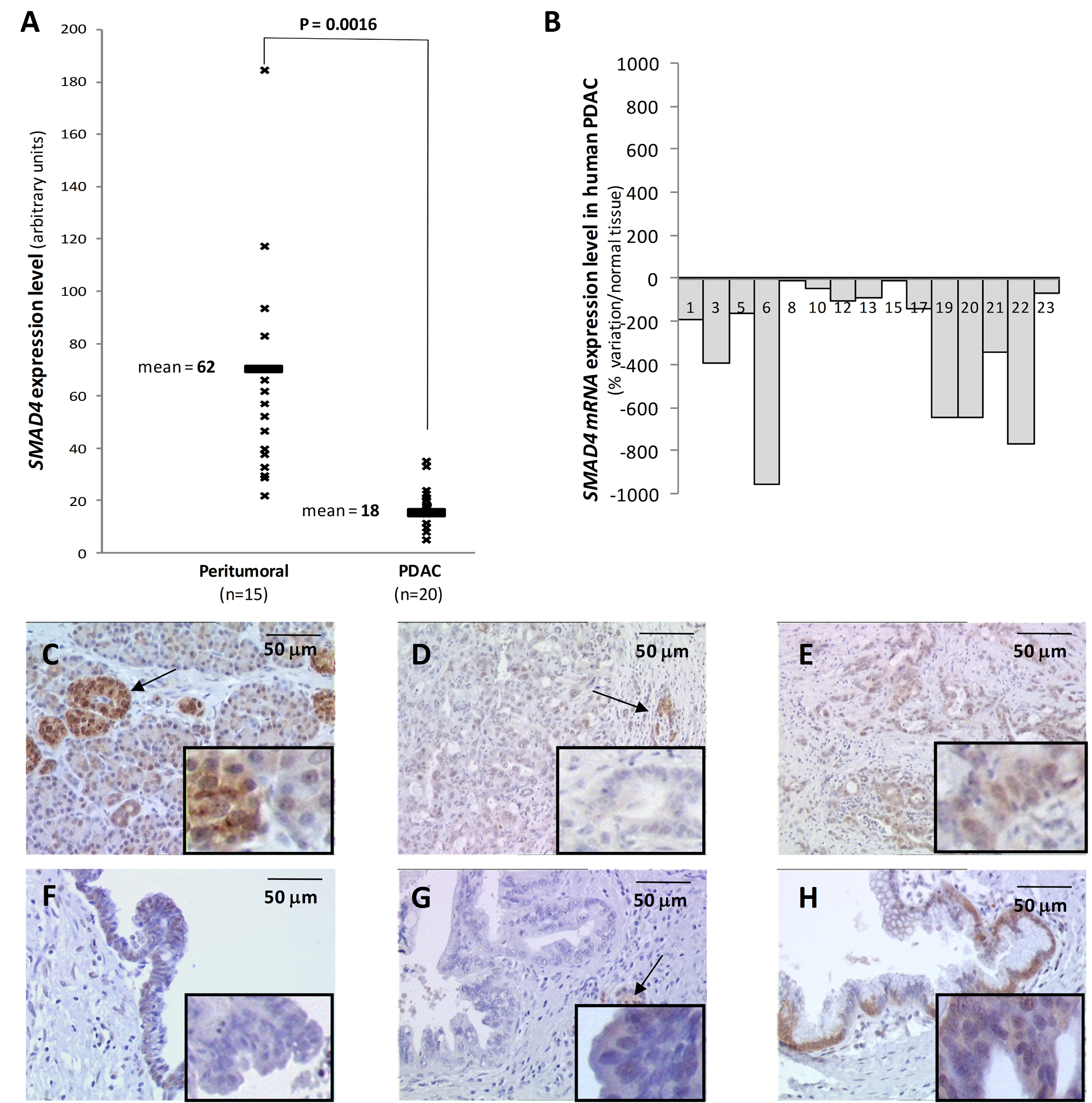

Supplement: Figure S4 — SMAD4 expression in human pancreatic neoplasia. Quantitative RT–PCR to detect SMAD4 expression from the 16 PDAC for which peritumoral tissue was available (A). For each patient represented by an individual bar, SMAD4 expression is represented as a percentage of variation relative to mRNA expression in the peritumoral tissue from the same patient (B). SMAD4 protein expression pattern was also assessed by immunohistochemistry. In the normal pancreas (C), SMAD4 is strongly detected in endocrine islets, a faint labeling is visible in acinar cells and ductal cells. In adenocarcinomas (n patients = 20), no labeling for SMAD4 was detected whereas adjacent residual endocrine cells are positive (D). In another example of adenocarcinoma, SMAD4 is faintly but readily detectable in neoplastic cells; the labeling is both cytoplasmic and nuclear (E). In IPMN grade 3, SMAD4 expression is heterogeneous; most cells are negative, while a few scattered cells retain a faint expression, usually nuclear (F). In PanIN grade 3, SMAD4 expression is either undetectable, as exemplified in the largest figure (note the persistent expression in adjacent residual endocrine cells (arrow)), or heterogeneous, as shown in the inset (G). In MCN, SMAD4 expression is usually strong, in low grade (large figure) as well as in high grade (inset) lesions (H). (3.34 MB TIF) [file pgen.1000575.s004.tif]
