# Supplementary material for: Inactivation of TIF1γ Cooperates with KrasG12D to Induce Cystic Tumors of the Pancreas
Source: PLoS Genet. 2009 Jul 24;5(7):e1000575. doi: 10.1371/journal.pgen.1000575 (PMC2706992; doi:10.1371/journal.pgen.1000575)
Supplement: Table S1 — Measurement of the area occupied by the normal pancreatic tissue. (0.06 MB DOC) [file pgen.1000575.s005.doc]

| **GENOTYPE** | **ID** | **Age (weeks)** | **Age (days)** | **Measured area (mm^2^)** | **Percentage of normal pancreatic tissue** |
| --- | --- | --- | --- | --- | --- |
| **Pdx1-Cre** ; **LSL-Kras^G12D^** ; **Tif1γ^lox/lox^** | #1 | **3** | 19 | 42 | **91 %** |
|  | #2 |  | 20 | 96 | **96 %** |
|  | #3 |  | 20 | 115 | **94 %** |
|  | #4 | **6-13** | 41 | 194 | **34 %** |
|  | #5 |  | 41 | 106 | **58 %** |
|  | #6 |  | 65 | UND | UND |
|  | #7 |  | 65 | UND | UND |
|  | #8 |  | 90 | 150 | **2 %** |
|  | #9 | **18-27** | 129 | 150 | **2 %** |
|  | #10 |  | 131 | 64 | **19 %** |
|  | #11 |  | 154 | 99 | **<1 %** |
|  | #12 |  | 189 | 91 | **44 %** |
| **Pdx1-Cre** ; **LSL-Kras^G12D^** | #13 | **4** | 30 | 59 | **>99 %** |
|  | #14 | **6-13** | 44 | 84 | **>99 %** |
|  | #15 |  | 68 | 45 | **>99 %** |
|  | #16 |  | 80 | 87 | **>99 %** |
|  | #17 | **18-27** | 125 | 83 | **99 %** |
|  | #18 |  | 159 | 81 | **91 %** |
|  | #19 | **51** | 356 | 53 | **83 %** |

**Table S1. Measurement of the area occupied by the normal pancreatic tissue**

Mice (ID, identity) from indicated genotypes were euthanized at different ages and their pancreas were analyzed by histology. We quantified the area occupied by the normal pancreatic tissue by excluding the regions devoid of acini as a consequence of the lesions (PanINs, IPMNs, area with massive inflammatory infiltrates). Normal pancreas is represented as a percentage of the whole measured pancreatic tissue. Measurements were done on several pictures taken from 2 non-contiguous pancreas sections. Quantification was done with AxioVision software. UND, undetermined.
